# Seasonal Variation in Essential Oil Compositions and Antioxidant Properties of *Acorus calamus* L. Accessions

**DOI:** 10.3390/medicines4040081

**Published:** 2017-11-08

**Authors:** Archana Parki, Pinky Chaubey, Om Prakash, Ravendra Kumar, Anil K. Pant

**Affiliations:** Department of Chemistry, College of Basic Sciences and Humanities, G.B. Pant University of Agriculture and Technology, Pantnagar U.S. Nagar-263145, Uttarakhand, India; parki.archana15@gmail.com (A.P.); pinkychaubey991@gmail.com (P.C.); ravichemistry.kumar@gmail.com (R.K.); anilpant54@gmail.com (A.K.P.)

**Keywords:** *Acorus calamus*, antioxidant activity, phenylpropanoids, asarone, essential oil

## Abstract

**Background:** *Acorus calamus* (Sweet flag) is a known herbal drug commonly used in traditional medicine. Our aim was to perform seasonal and altitudinal phytochemical screening to assess the antioxidant activity of the essential oils in the rhizome and leaves of *A. calamus* from three different altitudes. **Methods:** Phytochemical screening was performed using GC/MS analysis and in vitro antioxidant assay was done by different methods. **Results:** The essential oils mainly contained *α*-asarone, *β*-asarone (35.3–90.6%), and *Z*-isoelemicin (1.7–7.3%) as the major constituents, besides linalool, *Z*-methyl isoeugenol, shyobunone, kessane, etc. All the oils exhibited vast molecular diversity in terms of quantitative ingredients. All essential oils were studied for their antioxidant activity by different methods, including their effect on the DPPH radical-scavenging activity, reducing power, and chelating properties of Fe^2+^. The oils isolated in all the different seasons exhibited antioxidant activity as a function of concentration, with IC_50_ values ranging from 475.48 ± 0.08 to 11.72 ± 0.03 compared to standards. **Conclusion:** From the results obtained it can be inferred that the herb may be a good source of bioactive compounds and can work as an antioxidant to prevent oxidative deterioration in food. The data provide a basis for its in-situ investigation for judicious exploitation.

## 1. Introduction

*Acorus* is a genus of wetland monocot flowering plants distributed in North America and northern and eastern Asia, and naturalised in southern Asia and Europe from ancient cultivation [1,2,3,4,5,6]; they grow as herbs with perennial tuberous thick rhizomes in wetlands, particularly marshes [7]. The species are used in traditional medicine for the treatment of epilepsy, mental ailments, chronic diarrhoea, dysentery, bronchial catarrh, intermittent fevers, and glandular and abdominal tumours [8]. Plant diversity has considerable importance as a source of pharmaceutically active substances [9]. The natural antioxidants from plants can protect the human body from the attack of free radicals and retard the progress of many chronic diseases [10,11]. Natural antioxidants are generally classified as phenols, including flavonoids, phenolic acids and volatile compounds [7]. *Acorus calamus* is a traditional indigenous herb generally used in the treatment of cough, bronchitis, gout, tumours, haemorrhoids, skin diseases, numbness, and general debility [12,13]. It possesses a wide range of pharmacological activities, such as anti-diabetic [14], central nervous system depressant [15], anti-inflammatory [16], antioxidant [17], antispasmodic [18], antibacterial [19], antifungal [20], and cardiovascular [21] and insecticidal agent [22]. It has been reported by different workers that medicinal plants show a remarkable variation of active ingredients during different seasons; this is widely attributed to variations in environmental variables such as temperature and rainfall [23,24]. Besides its uses in traditional medicine, *A. calamus* has been used for digestive problems such as gas, bloating, colic, and poor digestive function; because of its rich ethnobotanical history, the herb is also used in the Ayurveda and Uniani systems of medicine. A number of bioactive constituents, viz., 2-allyl-5-ethoxy-4-methoxyphenol, 4-terpineol, lysidine, epieudesmin, spathulenol, furylethyl ketone, borneol, nonanoic acid, 2,2,5,5-tetramethyl-3-hexanol, galgravin, bornyl acetate, retusin, (9*E*,12*E*,15*E*)-9,12,15-octadecatrien-1-ol, geranylacetate, butyl butanoate, sakuranin, camphor, acetic acid, isoelemicin, acetaphenone, α-ursolic acid, dehydroabietic acid, methyl ether, isoeugenol, apigenin 4,7-dimethylether, linalool, dehydrodiisoeugenol, elemicin and linolenic acid, 1 beta,7 alpha(*H*)-cadinane-4 alpha,6 alpha,10 alpha-triol (1), 1 alpha,5 beta-guaiane-10 alpha-*O*-ethyl-4 beta,6 beta-diol (2), and 6 beta,7 beta(*H*)-cadinane-1 alpha,4 alpha,10 alpha-triol (3) have been reported in *A. calamus* [25,26,27]. The phenyl propanoids, sesquiterpenes, monoterpenes, xanthone glycosides, flavones, lignans, and steroids from *Acorus calamus* have been reported to possess various pharmacological activities such as insecticidal, larvicidal, antibacterial, mutagenic, cytotoxic, hepatoprotective, anticonvulsant, neuroleptic, smooth muscle relaxant, and smooth muscle stimulant activity [28]. The plant *A. calamus* has also been reported to be used in treating central nervous system abnormalities and normalizing the appetite, and various pharmacological activities like hepatoprotective, antidiabetic, antiproliferative, immunosuppressive, antidiarrhoeal, hypolipidemic, anti-spasmodic, and anti-proliferative have been reportedin extracts [29,30].

The essential oil composition of *A. calamus* rhizomes and concomitant antibacterial and antihelmintic activity has already been reported by our group [31,32]. However keeping in mind the above statements, the present study reports the results obtained on the seasonal and altitudinal diversity of essential oil components among three accessions of *A. calamus* from the Himalayan region of Uttarakhand in India. Publishing these data will help to generate a database of this plant and facilitate its more judicious and scientific exploitation in the future.

## 2. Material and Methods

### 2.1. Plant Material

Fresh samples of leaves and rhizomes of *A. calamus* were collected in different seasons (during 2014/2015) from three different altitudes of the Uttarakhand Himalayas in India. The plant was identified by Dr. D.S Rawat, a plant taxonomist at the Department of Biological Sciences, G.B., Pant University of Agriculture and Technology, Pantnagar, Uttarakhand, India. The GPS coordinates of the three locations were: INDIA, Uttarakhand, Pithoragarh district, Pithoragarh near Govt PG College, along stream, 29°34′59.17″ N, 80°11′49.46″ E, 1570 m a.s.l., July 2014, *Archna Parki s.n.*, GBPUH specimen Acc. No. 910. Naini Tal district, Bhimtal, along stream, 29°20′58.61″ N, 79°33′14.53″ E, 1340 m a.s.l., July 2014, *Archna Parki s.n.*, GBPUH Acc. No. 911 and Udham Singh Nagar district, near CBSH Pantnagar, in drainage channel, 29°01′27.2″ N, 79°29′27.2″ E, 236 m a.s.l., July 2014, Archana Parki *s.n.*, GBPUH Acc. No. 912.

### 2.2. Extraction of Essential Oils

Fresh leaves/rhizomes of *A. calamus* were collected from their natural habitat and tested in the phytochemistry research lab. To extract essential oil, the plant material (500 g) was crushed and separately subjected to hydro-distillation in Clevenger apparatus for 3 h using the apparatus described in the *European Pharmacopoeia* [33].

### 2.3. Gas Chromatography and Gas Chromatography/Mass Spectrometry

GC and GC/MS analyses of all the oils were performed on a Thermo Fischer GC apparatus using an DB5-5MS fused-silica capillary column (30 m × 0.25 mm i.d., 0.25 μm film thickness) equipped with a flame ionization (FID) detector. GC/MS analysis of the different essential oil samples were performed using a GC MS-QP 2010, in the following conditions. Column DB-5 (30 m × 0.25 mm i.d.; 0.25 μm film thickness; J&W Scientific, Agilent, Santa Clara, CA, USA); carrier gas: helium, with a flow rate of 1 mL/min; injection temperature: 250 °C; oven temperature programme: initial temperature 80 °C, isothermal for 2 min, RAMP 7 °C/min, final temperature 280 °C, and isothermal for 10 °C/min. Ionization mode: EI (70 eV), mass range: 40–6500 amu. The compounds were identified with the help of NIST-MS, FFNSC Wiley Library, and comparing the data with literature reports and GC retention indices [34].

### 2.4. Antioxidant Activity

To analyse the in vitro antioxidant property, the essential oils of *A. calamus* were subjected to the following methods.

#### 2.4.1. DPPH Radical Scavenging Activity

This is a quick method to study the scavenging ability of the antioxidants [35]. Briefly, the tested samples (5–25 μL/mL) were added to 5 mL of a 0.004% methanol solution of DPPH. Finally, the absorbance was read against a blank at 517 nm after 30 min of incubation at room temperature. Ascorbic acid was used as the standard antioxidant. Inhibition of free radical by DPPH in percent (IC %) was calculated using the equation. IC% = (A_0_ − A_t_)/A_0_ × 100, where A_0_ = the absorbance value of the control sample, A_t_ = the absorbance value of the test sample, and IC = inhibitory concentration. The radical scavenging activities of essential oils were discussed in terms of their IC_50_ values.

#### 2.4.2. Reducing Power

The reducing power of essential oils was determined by the method developed earlier [36]. In brief, varying concentrations of tested samples (5–25 μL/mL) were mixed with 2.5 mL of phosphate buffer (200 mM, pH = 6.6) and 2.5 mL of 1% potassium ferricyanide, K_3_ [FeCN_6_]. The mixtures were incubated for 20 min at 50 °C. After incubation, 2.5 mL of trichloroacetic acid was added to the mixtures, followed by centrifugation at 650 rpm for 10 min. The upper layer (1 mL) was mixed with 5 mL distilled water and 1 mL of 0.1% ferric chloride and the absorbance of the resultant solution was measured at 700 nm. Reducing power % = (A_0_ − A_t_)/A_0_ × 100, where A_0_ = the absorbance value of the control sample and A_t_ = the absorbance value of the test sample. The percent of chelating ability was plotted against concentrations and using a standard (gallic acid). The reducing potential of essential oils was discussed in terms of their RP_50_ values.

#### 2.4.3. Metal Chelating Activity

The chelation of Fe^2+^ by essential oils was evaluated using the method developed earlier [37]. In brief, 0.1 mL of 2 mM FeCl_2_·4H_2_O, 0.2 mL of 5 mM ferrozine, and 4.7 mL of methanol were added to different concentrations of a test sample (5–25 μL/mL). The solutions were mixed and allowed to react for 10 min. The absorbance was determined at 562 nm; IC% = (A_0_ − A_t_)/A_0_ × 100, where A_0_ = the absorbance value of the control sample and A_t_ = the absorbance value of the test sample. The percent of chelating ability was plotted against the concentration, and the standard curve was drawn using a standard antioxidant (EDTA). The metal chelating ability of essential oils was discussed in terms of their IC_50_ values.

## 3. Results and Discussion

A noticeable variation was observed in the percentage yield of the hydro distilled essential oils (EOs) samples, taken at four seasons (winter, spring, summer, and autumn), The essential oil was pale yellow with a characteristic odour, and produced an irritating sensation in the eyes. The yields of essential oils in different seasons were 0.02–1.3% for leaves and 1.2–4.8% *w*/*v* for rhizomes. However, in previous reports the yields of essential oils in *A. gramineus* and *A. calamus* from different regions have been reported to range from 1.0 to 3.5% [31,38,39]. In the present study, the yield showed the highest percentage (4.8%) during the summer in all accessions, whereas during autumn and winter the yields obtained were only up to 0.02%.

**Figure 1:**
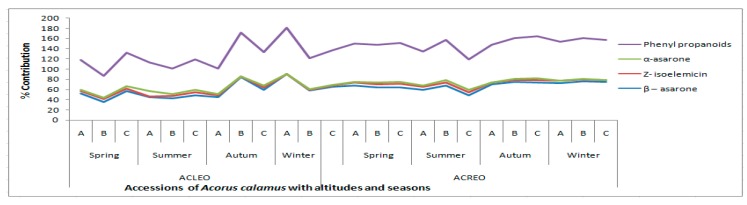
Phenylpropanoids in ACLEO and ACREO in different seasons from different altitudes.

GC/MS showed marked variation in the major ingredients of oils prepared in spring, summer, autumn, and winter, respectively. The composition of the essential oils differed quantitatively and qualitatively according to the time of collection. A detailed comparative analysis of all the oils with different seasons has been recorded in Table 1, with phenyl propanoids in Table 2 and Figure 1, while the class composition is in Table 3 and Table 4.

**Figure 2:**
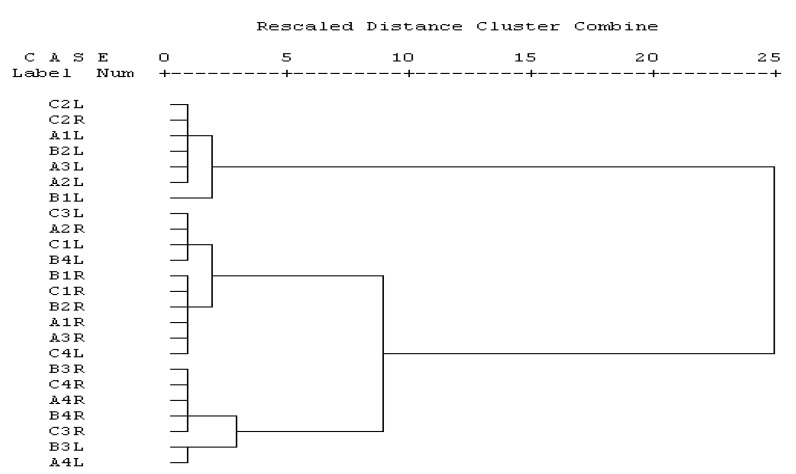
Dandrogram of major compounds (phenyl propanoids) in ACLEO and ACREO prepared Software SPSS, ward method with square Euclidean distance measure. Where 1 = spring, 2 = summer, 3 = autumn, 4 = winter seasons, L = *A. calamus* leaves essential oils (ALEO), R = *A. calamus* rhizomes essential oils (RLEO).

The oils were dominated by phenylpropanoids, and were detected in all the accessions in all seasons but in different quantities. Interestingly, the *β*-asarone content was highest in winter (57.0–90.6%) and lowest in summer, except Bhimtal, in which spring has the lowest content; whereas the *α*-asarone content was highest in summer and lowest in winter in all the accessions. On the basis of major components (phenyl propanoids) analysis by cluster analysis, as in Figure 2, it was observed that there were little difference in principal components like *α*-asarone, *Z*-isoelemicin, *β*-asarone, etc., both altitude-wise and season-wise.

Over 78–96% of constituents were identified in both ACLEO and ACREO. The major constituents identified in both the oils in all four seasons were *trans*-methyl isoeugenol, *Z*-isoelimicin, *α*-asarone, *β*-asarone, etc., but with different respective yield. Both ACLEO and ACREO exhibited similar qualitative diversity in terms of terpenoid composition, but their quantity varied in different seasons. However, the constituents, viz., limonene, *α*-humulene, 2,4,5-trimethoxy benzoic acid, and heptadecanol, could be detected only in ACLEO, whereas elimicin and *β*-calacorene were detected only in ACREO.

The seasonal variation in the chemical composition of essential oils might be due to different altitudes, environmental conditions, and the developmental stage/season of the plant materials, which is in agreement with results reported earlier [40,41,42]. In terms of class composition, the monoterpenoids in ACLEO from all three accessions in four different seasons ranged from 0.2 to 15.7%, mainly represented by limonene, *β* oscimene, linalool, bornyl acetate, etc. The sesquiterpenoids ranged from 2.6 to 16.6% with E caryophyllene, *β* elemene, *α* copaene, calarene, *Z* methyl isoeugenol, *α* humulene, spathulenol, germacrene D, *α* cadenene, *α* calacorene, etc. The marker class of, phenyl propanoids, ranged from 36.9 to 90.8%, mainly represented by *α* asarone, *β* asarone, *Z* isoelimicin, elimicin, etc. (Table 2 and Table 3). Similarly, in ACREO the monoterpenoids ranged from 0.1 to 9.5%, and sesquiterpenoids from 0.6 to 9.4%, while phenyl propanoids ranged from 60.8 to 82.4% in three accessions for the four seasons. The essential oil composition of rhizomes from our laboratory has already been reported. The essential oil was found to possess significant antibacterial and antihelmintic activity [31,32]. It has also been reported that asarone and sesquiterpenoids were the major constituents in two phylogenetically different accessions [38]. Patra and Mitra [43] and Tamas et al. [44] have also reported acoramone and phenylpropane derivatives like *α*-asarone, *β*-asarone, *γ*-asarone, isoeugenol, and methyl ether in essential oils. The components reported earlier were also identified, besides some other constituents, but in different quantities. Seasonal along with altitudinal variation in both rhizomes (ACREO) and the aerial part (ACLEO) are reported here for the first time, demonstrating the qualitative and quantitative diversity of constituents in the essential oils of *A. calamus.*

All the essential oils isolated in different seasons exhibited DPPH radical scavenging activity in a dose-dependent manner (5 µL/mL–25 µL/mL) (Table 5). The radical scavenging potential of ACREO and ACLEO from three altitudes in the form of their IC_50_ values in four different seasons was observed as Spring: ACBTREO > ACPGLEO > ACBTLEO > ACPNLEO > ACPNREO > ACPGREO; Summer: ACPNREO > ACPGREO > ACBTREO > ACPGLEO > ACPNLEO > ACBTLEO; Autumn: ACPNREO > ACPNLEO > ACBTREO > ACPGLEO > ACBTLEO > ACPGREO; Winter: ACPNREO > ACBTREO > ACPNLEO > ACPGLEO > ACPGREO > ACBTLEO, respectively, compared to the standards, BHT (IC_50_ = 28.37 µg/mL) > catechin (IC_50_ = 28.48 µg/mL). The antioxidant power of EOs might be attributed to their hydrogen donating ability to DPPH free radicals.

In terms of Fe^3+^ reducing activity at selected dose levels of 5–25 µg/mL, the EOs exhibited dose dependent reducing power activity. The RP_50_ values in different seasons for all the EOs were observed in the order of Spring: ACPGREO > ACPGLEO > ACBTREO > ACBTLEO > ACPNLEO > ACPNREO; Summer ACBTREO > ACPGREO > ACPNREO > ACBTLEO > ACPNLEO > ACPGLEO; Autumn ACPGREO > ACBTREO > ACPNREO > ACBTLEO > ACPNLEO > ACPGLEO; Winter ACPGREO > ACBTREO > ACBTLEO > ACPNLEO > ACPNREO > ACPGLEO. However, the RP_50_ of standards were observed in the order gallic acid (RP_50_ = 56.07 µg/mL) > catechin (RP_50_ = 71.74 µg/mL) (Table 5).

Similarly, the dose-dependent response for chelating activity for all the Eos (Table 5) exhibited the following order of IC_50_ values in different seasons: Spring ACPGLEO > ACPNLEO > ACBTLEO > ACPGREO > ACBTREO > ACPNREO; Summer ACPNLEO > ACPGLEO > ACBTLEO > ACPNREO > ACPGREO > ACBTREO; Autumn ACPNLEO > ACPGLEO > ACBTLEO > ACPNREO > ACBTREO > ACPGREO; and Winter ACBTREO > ACBTLEO > ACPGREO > ACPGLEO > ACPNREO. The IC_50_ for standard EDTA and citric acid were IC_50_ = 38.74 µg/mL and 45.57 µg/mL, respectively, under the same experimental conditions (Table 5).

The EOs from *A. calamus* exhibited good in vitro antioxidant activity, which might be because of the mixture of essential oils containing mono and sesquiterpenoids and the synergetic effects of the constituents. This is proven by a report that says that antioxidant capacity is affected by other bioactive compounds and could involve synergistic effects [45]. Several reports have shown in vitro antioxidant properties of many natural products, including essential oils. Antioxidants are believed to be directly anti mutagenic. In vitro physicochemical assays characterize most of them as antioxidants. However, the published report shows that in eukaryotic cells, essential oils can act as pro-oxidants, affecting inner cell membranes and organelles like mitochondria, and are usually non-genotoxic, and hence the beneficial effects of essential oils are due to pro-oxidants’ effects on the cellular level [46]. The anticancer and antioxidant properties of certain medicinal herbs are used to treat trauma over a longer period of time, which is promising. *Acorus calamus* extracts and essential oils have been reported to possess anticancer and anti-angiogenic effects on cancer cells [47,48,49], which might be due to its antioxidant activity. In folk medicine the herb *A. calamus* has been used as a wound healing agent for many years, which has been proven scientifically by reporting the significant wound-healing activity of aqueous extracts in the animal model of excise wound healing, and anti-inflammatory activity in vitro [50].

It has been reported that essential oils containing linalool and the corresponding acetate play a major role in terms of anti-inflammatory activity [51]. The compounds shyobunone and isoshyobunone, isolated from essential oils, have been reported to possess insecticidal and repellant activity against *Lasioderma serricorne* (LS) and *Tribolium castaneum* (TC) [52]. Z methyl isoeugenol is used in perfumes as a flavouring agent [53]. Elimicin and caryophyllene have been reported to possess anti-inflammatory, antimicrobial, and analgesic activity [54,55,56]. Palmitic acid, regardless of obesity, impairs leptin and insulin’s ability to regulate food intake and body weight. In addition, it has been reported that fatty acids containing palmitic acid and its ester possess significant antifungal and antibacterial activity [57,58]. Various essential oil components present in essential oils like linalool, 1,8-cineol, caryophyllene, α humulene, and asarone have been reported to possess antioxidant activity [59,60,61]. Components like linalool, asarone, *α* humulene, and caryophyllene oxide are also present in essential oils. Based on the reported data, it can be inferred that the antioxidant activity of *A. calamus* essential oil is because of these compounds, besides the synergetic effects of other constituents in the oil.

## 4. Conclusions

On the basis of our results, we can conclude that seasonal fluctuations periodically impact on the production of the constituents in medicinal plants, and also likely influence their therapeutic efficiency. Our study reveals that *A. calamus* plants show a rhythmic increase in oil production throughout the growing season and decline towards the winter. Hence, late summer can be the best time for collecting *A. calamus* plants. The present study reveals the presence of bioactive compounds, the antioxidant activity, and the free radical scavenging activity of *A. calamus*. Thus, this study supports the use of *A. calamus* against various ailments. More bioactive compounds present in EOs of *A. calamus* may warrant further characterisation.

## Figures and Tables

**Table 1:** Comparative chart on seasonally GC/MS analysis of *Acorus calamus* leaves and rhizome essential oils.

S. N.	Compounds	RI	ACLEO												ACREO											
			SEASONS																							
			SPRING			SUMMER			AUTUMN			WINTER			SPRING			SUMMER			AUTUMN			WINTER		
			Sites of Collection												Sites of Collection											
			A	B	C	A	B	C	A	B	C	A	B	C	A	B	C	A	B	C	A	B	C	A	B	C
1	bornyl acetate	948	-	0.1	-	-	1.0	-	-	-	-	-	-	-	-	-	-	-	0.1	-	-	-	-	-	-	-
2	limonene	1031	0.8	-	-	-	0.2	0.2	0.1	t	0.5	1.9	1.3	0.8	-	-	-	-	-	-	-			-	-	-
3	trans-β ocimene	1050	-	t	-	-	0.2		0.1	t	-	1.3	3.0	0.8	0.5	0.9	-	1.9	2.0	0.1	0.5	-		1.2	1.9	0.3
4	linalool	1096	9.2	5.8	0.9	0.5	7.2	6.4	3.2	0.7	7.3	1.9	5.6	2.6	0.6	0.8	-	1.8	1.5	6.4	0.3	0.5	0.2	0.8	0.4	-
5	shyobunone	1324	9.3	7.6	5.0	0.4	7.0	6.6	7.1	2.5	6.0	-	6.9	8.8	4.4	4.2	4.7	5.1	3.6	6.6	3.3	2.7	2.8	5.5	4.5	8.0
6	α-copaene	1376	-	t	-	-	0.2	-	-	-	-	-	-	-	-	t	-	-	0.1	-	-	t	-	-	-	-
7	β elemene	1390	-	-	-	0.2	0.1	-	-	0.2	-	-	-	-	0.1	0.1	-	0.1	0.1	0.6	-		-	-	-	-
8	aristolene	1416	-	2.0	-	-	-	-	-	-	-	-			1.1	0.4	1.0	1.6	-	-	2.0	-	0.2	1.0	0.6	0.7
9	E caryophyllene	1418	2.0	1.8	1.9	0.4	1.7	-	3.1	0.5	2.0	-	2.0	1.8	-	-	-	-	-	1.6	-	-	-	-	-	-
10	calarene	1432	-			-	-	-	-			-	8.2		2.3	0.7	1.9	2.9	0.5	-	4.2	0.6	0.4	2.2	0.9	1.9
11	α-humulene	1440	0.7	1.1	0.5	0.2	0.8	0.5	1.4	0.2	0.3	-	0.9	0.6	-	-	-	-	-	-	-	-	-	-	-	-
12	α-muurolene	1478	-	-	0.2	-	0.1	0.1	0.3	-	-	-	-	-	-	-	0.1	-	-	0.1	-	-	-	-	1.0	-
13	germacrene D	1480	0.4	0.1	0.4	-	0.6	0.5	0.3	-	0.3	-	0.3	0.2	-	0.1	-	-	-	0.5	-	-	-	-	-	0.1
14	*Z-*methyl isoeugenol	1492	2.8	2.8	2.2	0.4	2.3	1.6	3.3	0.5	2.1	-	2.2	4.7	5.1	3.4	3.8	6.3	4.6	1.6	5.0	9.0	4.1	7.4	4.5	4.9
15	viridiflorene	1496	-	0.4	1.2	0.1	1.4	1.5	0.9	-	-	-	-	-	-	-	-	-	-	1.5	-	-	-	-	-	-
16	dehydroxy-isocalamendiol	1497	6.1	-	-	-	-	-	-	-	-	-	-	4.8	-	-	-	-	-	-	-	-	2.3	-	-	1.4
17	δ-cadinene	1524	0.3	1.1	1.1	0.3	1.3	0.3	0.8	0.3		-	-	-	0.4	0.4	0.3	0.6	0.3	-	-	0.3	-	-	0.4	0.2
18	kessane	1528	1.0	3.2	0.1	-	2.1	0.1	2.0	0.3	0.3	-	1.9	0.5	0.7	0.5	0.3	1.5	0.4	0.1	0.2	-	-	0.5	0.5	
19	α-cadinene	1538	-	1.0		-	-	-	0.2	-	-	-	-	-	-	-	-	-	-	0.3	-	-	-	-	-	-
20	α-calacorene	1545	-	0.2	0.2	-	0.2	-	0.2	-	-	-	-	-	0.6	0.2	0.4	0.3	0.1	-	-	0.5	-	-	-	-
21	α-elemol	1547	-	-	-	-	0.7	-	0.7	0.1	-	-	-	-	-		-	-	-	-	-	-	-	-	-	-
22	β-calacorene	1548	-	-	-	-	-	-	-	-	-	-	-	-	0.1	0.1	0.1	0.1	0.	-	-	-	-	-	-	-
23	elemicin	1554	-	-	-	0.2	-	-	-	2.1	-	-	-	-	0.9	1.2	-	0.9	1.6	1.0	-	0.7	-	-	0.6	-
24	spathulenol	1575	-	0.4		0.1			-	-		-			-	-	-	-	-	-	-	-	-	-	-	-
25	caryophyllene oxide	1581	-	-	1.3		1.0		0.5	-	-	-	-	-	-	-	-	-	-	-	-	-	-	-	-	-
26	β-asarone	1617	52.2	35.3	56.5	44.4	42.4	48.6	44.6	84.3	59.4	90.6	57.6	65.2	68.0	64.4	64.3	59.5	67.7	48.6	70.0	74.5	73.7	73.1	76.6	74.9
27	asaronaldehyde	1620	-	4.3	0.6	0.9	2.9	0.1	2.1	-	-	-	-	-	0.2	1.0	0.6	-	-	0.1	-	-		-	-	-
28	*Z*-isoelemicin	1644	4.5	6.3	5.5	1.8	4.8	6.0	4.1	1.7	4.6	-	2.5	3.0	5.7	6.0	7.3	5.5	6.7	6.0	3.8	4.6	5.0	4.0	3.3	3.4
29	α-asarone	1676	2.0	1.8	4.3	10.5	3.7	5.2	2.2	-	3.1	0.2	0.7	0.2	1.3	3.4	3.9	2.7	4.4	5.2	0.3	1.5	3.7	0.1	0.7	0.8
30	2,4,6-trimethoxyacetophenone 3 methyl	1701	-	0.4	0.6	-	-	-	0.1	-	-	-	-	-	-	-	-	-	-	-	-	-	-	-	-	-
31	aspidinol	1833	-	-	-	0.4	-	-	1.4	-	-	-	-	-	-	-	-	-	-	-	-	-	-	-	-	-
32	2,4,5-trimethoxybenzoic acid	1927	-	2.3	0.6	-	-	-	1.1	-	-	-	-	-	-	-	-	-	-	-	-	-	-	-	-	-
33	phytol	1941			2.3	2.8		1.1	0.6		0.8			0.4	-	-	-	-	-	1.2		-	-	-	-	-
34	palmitic acid	1984	-	-	-	13.3	-	-		-	-	-	-	-	-	-	-	-	-	-	-	-	-	-	-	-
**total**			**91.3**	**78**	**85.4**	**76.9**	**81.9**	**78.8**	**80.4**	**93.4**	**86.7**	**95.9**	**93.1**	**94.4**	**93.4**	**88.8**	**88.7**	**89.4**	**92.7**	**81.5**	**92.7**	**94.9**	**92.4**	**92.7**	**95.9**	**96.6**
**SD (±)**			9.2	6.3	10.0	8.0	7.5	8.7	7.7	14.9	10.6	15.7	10.3	11.7	11.6	11.1	11.0	10.2	11.6	8.4	12.2	13.2	13.2	12.5	13.1	13.1

T = trace (0.05%), A = Pithoragarh location (hilly region), B = Bhimtal location (sub hilly region), C = Pantnagar location (tarai region), ACLEO = *A. calamus* leaves essential oil, ACREO = *A. calamus* rhizome essential oil ACREO.

**Table 2:** Phenylpropanoids in ACLEO and ACREO in different seasons from different altitudes.

SN	Phenyl Propanoids	RI	ACLEO												ACREO											
			SEASONS																							
			SPRING			Summer			AUTUMN			WINTER			SPRING			SUMMER			AUTUMN			WINTER		
			Sites of Collection												Sites of Collection											
			A	B	C	A	B	C	A	B	C	A	B	C	A	B	C	A	B	C	A	B	C	A	B	C
1	β-asarone	1617	52.2	35.3	56.5	44.4	42.4	48.6	44.6	84.3	59.4	90.6	57.6	65.2	68.0	64.4	64.3	59.5	67.7	48.6	70.0	74.5	73.7	73.1	76.6	74.9
2	Z-isoelemicin	1644	4.5	6.3	5.5	1.8	4.8	6.0	4.1	1.7	4.6	-	2.5	3.0	5.7	6.0	7.3	5.5	6.7	6.0	3.8	4.6	5.0	4.0	3.3	3.4
3	α-asarone	1676	2.0	1.8	4.3	10.5	3.7	5.2	2.2	-	3.1	0.2	0.7	0.2	1.3	3.4	3.9	2.7	4.4	5.2	0.3	1.5	3.7	0.1	0.7	0.8
4	Phenyl propanoids		58.7	43.4	66.3	56.7	50.9	59.8	50.9	86	67.1	90.8	60.8	68.4	75	73.8	75.5	67.7	78.8	59.8	74.1	80.6	82.4	77.2	80.6	79.1

A = Pithoragarh location (hilly region), B = Bhimtal location (sub hilly region), C = Pantnagar location (tarai region), ACLEO = *A. calamus* leaves essential oil, ACREO *= A. calamus* rhizome essential oil ACREO.

**Table 3:** Classes of compounds identified in ACLEO from four seasons.

Class	SPRING			SUMMER			AUTUMN			WINTER		
	A	B	C	A	B	C	A	B	C	A	B	C
Hydrocarbons	[4.2]	[7.7]	[6.8]	[1.2]	[7.8]	[3.1]	[7.9]	[4.2]	[3.1]	[3.2]	[15.7]	[4.2]
Monoterpenoids	0.8	-	-	-	0.4	0.2	0.2	-	0.5	3.2	4.3	1.6
Sesquiterpenoids	3.4	7.7	6.8	1.2	7.4	2.9	7.7	4.2	2.6	-	11.4	2.6
Oxygenated compounds	[28.4]	[26.9]	[12.3]	[18.8]	[23.2]	[15.9]	[21.6]	[8.1]	[16.5]	[1.9]	[16.6]	[21.8]
Monoterpenoids	12	15.7	4.9	2.2	13.4	8.1	11.2	5.2	9.4	1.9	7.8	7.3
Sesquiterpenoids	16.4	11.2	7.4	16.6	9.8	7.8	10.4	2.9	7.1		8.8	14.5
Phenyl propanoids	58.7	43.4	66.3	36.9	50.9	59.8	50.9	58.6	67.1	90.8	60.2	68.4

A = Pithoragarhlocation (hilly region), B = Bhimtal location (sub hilly region), C = Pantnagar location (tarai region).

**Table 4:** Classes of compounds identified in ACREO from four seasons.

Class	SPRING			SUMMER			AUTUMN			WINTER		
	A	B	C	A	B	C	A	B	C	A	B	C
Hydrocarbons	[5.1]	[2.9]	[3.8]	[7.5]	[3.1]	[4.7]	[6.7]	[1.4]	[0.6]	[4.4]	[4.8]	[3.2]
Monoterpenoids	0.5	0.9		1.9	2.0	0.1	0.5			1.2	1.9	0.3
Sesquiterpenoids	4.6	2.0	3.8	5.6	1.1	4.6	6.2	1.4	0.6	3.2	2.9	2.9
Oxygenated compounds	[11.0]	[9.9]	[9.4]	[14.7]	[10.2]	[16.0]	[8.8]	[12.2]	[9.4]	[14.2]	[9.9]	[14.3]
Monoterpenoids	5.9	5.2	4.4	8.1	6.2	8.1	5.3	9.5	4.3	8.2	4.9	4.9
Sesquiterpenoids	5.1	4.7	5.0	6.6	4.0	7.9	3.5	2.7	5.1	6.0	5.0	9.4
Phenyl propanoids	75.9	75	75.5	68.6	80.4	60.8	74.1	81.3	82.4	77.2	81.2	79.1

A = Pithoragarhlocation (hilly region), B = Bhimtal location (sub hilly region), C = Pantnagar location (tarai region).

**Table 5:** Antioxidant activity in terms of IC_50_ values for leaves and rhizome essential oils.

S.N.	Sample Name	DPPH IC_50_ Value (µg/mL) ± SD				Reducing RP_50_ Value (µg/mL) ± SD				Chelating IC_50_ Value (µg/mL) ± SD			
		Seasons											
		**SPRING**	**SUMMER**	**AUTUMN**	**WINTER**	**SPRING**	**SUMMER**	**AUTUMN**	**WINTER**	**SPRING**	**SUMMER**	**AUTUMN**	**WINTER**
1	**ACPGREO**	198.06 ^h^ ±0.07	41.46 ^c^ ±0.66	107.52 ^g^ ±3.96	78.03 ^f^ ±3.30	137.14 ^b^ ±1.39	72.89 ^b^ ±0.01	52.30 ^a^ ±3.99	65.32 ^a,b^ ±0.15	34.82 ^c^ ±0.27	68.53 ^a^ ±0.99	206.75 ^e^ ±4.30	24.00 ^c^ ±0.12
2	**ACPGLEO**	59.20 ^d^ ±0.36	59.36 ^f^ ±0.81	83.07 ^e^ ±2.73	70.36 ^e^ ±2.80	168.97 ^b^ ±0.89	320.11 ^e^ ±2.47	227.63 ^d^ ±4.36	350.98 ^f^ ±3.88	15.24 ^a^ ±0.06	13.08 ^a^ ±0.13	158.10 ^b^ ±11.56	25.78 ^d^ ±0.12
3	**ACPNREO**	188.36 ^g^ ±3.59	41.35 ^c^ ±0.10	59.02 ^c^ ±1.26	37.31 ^c^ ±0.19	475.48 ^e^ ±28.19	77.72 ^b^ ±0.19	71.54 ^b^ ±0.12	325.83 ^e^ ±0.04	47.25 ^f^ ±0.32	41.29 ^b^ ±0.37	187.41 ^d,e^ ±10.59	30.04 ^f^ ±0.25
4	**ACPNLEO**	116.22 ^f^ ±3.14	78.71 ^g^ ±2.83	75.53 ^d^ ±2.11	58.71 ^d^ ±0.71	414.19 ^d^ ±17.8	245.77 ^d^ ±7.68	131.70 ^c^ ±1.25	201.25 ^d^ ±4.74	29.78 ^b^ ±0.22	11.72 ^c^ ±0.03	153.13 ^b^ ±13.55	26.94 ^e^ ±0.20
5	**ACBTREO**	49.67 ^c^ ±1.53	51.42 ^e^ ±0.65	82.52 ^e^ ±1.88	38.52 ^c^ ±0.62	326.87 ^c^ ±11.83	72.24 ^b^ ±0.18	71.08 ^b^ ±0.10	84.97 ^b^ ±0.22	44.98 ^e^ ±1.34	84.64 ^c^ ±2.25	193.54 ^e^ ±9.28	18.21 ^a^ ±0.03
6	**ACBTLEO**	83.94 ^e^ ±1.97	46.36 ^d^ ±0.94	96.17 ^f^ ±2.85	98.27 ^g^ ±4.44	382.27 ^d^ ±2.92	161.65 ^c^ ±0.82	73.98 ^b^ ±0.19	133.34 ^c^ ±18.88	30.88 ^b^ ±0.14	16.84 ^d^ ±0.59	164.46 ^b,c^ ±9.19	19.82 ^b^ ±0.07
7	BHT *	27.74 ^b^ ±0.05	27.74 ^b^ ±0.04	27.74 ^b^ ±0.04	28.37 ^a^ ±0.20	-	-	--	-	-	-	-	-
8	Catechin *	18.53 ^a^ ±0.15	18.53 ^a^ ±0.15	18.53 ^a^ ±0.15	28.48 ^b^ ±0.17	71.74 ^b^ ±1.39	71.74 ^b^ ±0.49	71.74 ^b^ ±0.49	71.74 ^a,b^ ±0.49	-	-	-	-
9	**Gallic acid**	-	-	-	-	56.14 ^b^ ±1.39	56.07 ^a^ ±0.91	56.07 ^a^ ±0.91	56.07 ^a^ ±0.91	-	-	-	-
10	**EDTA**	-	-	-	-	-	-	-	-	38.74 ^d^ ±0.33	38.74 ^e^ ±0.33	38.74 ^a^ ±0.33	38.74 ^g^ ±0.33
11	**Citric acid**	-	-	-	-	-	-	-	-	45.57 ^e^ ±0.33	45.57 ^f^ ±0.33	45.57 ^a^ ±0.33	45.57 ^h^ ±0.33

ACPGREO = *Acorus calamus* Pithoragarh rhizome essential oil, ACPGLEO = *Acorus calamus* Pithoragarh leaves essential oil, ACPNREO = *Acorus calamus* Pantnagar rhizome essential oil, ACPNLEO = *Acorus calamus* Pantnagar leaves essential oil, ACBTREO = *Acorus calamus* Bhimtal rhizome essential oil, ACBTLEO = *Acorus calamus* Bhimtal leaves essential oil. *-* = Not applicable, Values are means of three replicates ± SD, Within a column, mean values followed by the same letter are not significantly different according to Tukey’s test (*p* < 0.05).
